# Mechanisms underlying alterations of the gut microbiota by exercise and their role in shaping ecological resilience

**DOI:** 10.1093/femsre/fuaf037

**Published:** 2025-08-12

**Authors:** Alex E Mohr, Núria Mach, Jamie Pugh, Gregory J Grosicki, Jacob M Allen, J Philip Karl, Corrie M Whisner

**Affiliations:** College of Health Solutions, Arizona State University, Phoenix, AZ 85004, United States; Oak Ridge Institute for Science and Education, Oak Ridge, TN 37830, United States; Military Nutrition Division, U.S. Army Research Institute of Environmental Medicine, Natick, MA 01760, United States; Center for Health Through Microbiomes, Biodesign Institute, Arizona State University, Tempe, AZ 85281, United States; IHAP, Université de Toulouse, INRAE, ENVT, Toulouse, 31076, France; School of Sport and Exercise Sciences, Liverpool John Moores University, Liverpool, L3 5AH, United Kingdom; Biodynamics and Human Performance Center, Georgia Southern University, Savannah, GA 31419, United States; Department of Health and Kinesiology, University of Illinois at Urbana-Champaign, Urbana, IL 61801, United States; Military Nutrition Division, U.S. Army Research Institute of Environmental Medicine, Natick, MA 01760, United States; College of Health Solutions, Arizona State University, Phoenix, AZ 85004, United States; Center for Health Through Microbiomes, Biodesign Institute, Arizona State University, Tempe, AZ 85281, United States

**Keywords:** microbiome, athlete, acute stress, microbial ecology, precision health, gut–muscle axis

## Abstract

The gut microbiota (GM) is a dynamic ecosystem intricately linked to human health, including metabolic, immune, endocrine, and gastrointestinal functions. Exercise is recognized as a significant modifier of this microbial ecosystem, yet the complexities of this relationship are underexplored. Here, we delve into the multifaceted interactions between structured physical activity and the GM, emphasizing the role of exercise-induced stressors in shaping microbial composition and function. Unique to our review, we discuss the acute effects of different forms of exercise-induced stress on the GM and explore how these responses may influence long-term adaptability, stability, and resilience. Furthermore, we address critical junctures in microbial dynamics leading to shifts between different stable states. Finally, we explore the implications of host-controlled factors such as diet, exercise training, and nutritional supplementation in modulating the microbial community in the gut to optimize athletic performance. We conclude that while the potential to harness the synergistic effects of exercise-induced stressors, dietary interventions, and microbial adaptations appears promising, current evidence remains preliminary, highlighting the need for additional targeted research to guide future strategies that manipulate the GM for optimal health and athletic performance.

## Teaser

Exploring the complex interplay between exercise and the gut microbiota, this review highlights how structured physical activity acts as a hormetic stressor, altering gut microbial diversity and resilience, and shaping systemic health and athletic performance. Image created with BioRender.com.

## Introduction

The gut microbiota (GM), comprising bacteria, archaea, viruses, eukaryotes, and fungi within the gastrointestinal (GI) tract, plays a critical role in human health due to its complex genetic makeup and functional diversity. Advances in combinatorial multiomics research have enabled detailed analysis of the GM’s taxonomic structure, functions, and metabolic profiles, including the community’s development after birth (Chu et al. 2017), differences across health states (Turnbaugh et al. 2008), and utility for predictive disease modeling (Liu et al. 2022b). Viewed broadly within the systems biology framework, the GM responds to environmental changes created by interventions, behaviors, infections and diseases, and host factors (Fassarella et al. 2021). Indeed, taxonomic profiles of the GM in an adult human can change within days when challenged with extreme dietary alteration (David et al. 2013). Over longer periods, sustained adherence to specific dietary patterns may significantly shape these communities with implications for health outcomes (Fackelmann et al. 2025). Additionally, large perturbations such as antibiotic administration (Fishbein et al. 2023) or chronic stress (Franzosa et al. 2019) can move the ecosystem into different stable states—“healthy” or otherwise (Lozupone et al. 2012). However, the GM of adults also demonstrates resilience—determined by the ability to bend upon exposure to significant stress or perturbation (resistance) before breaking toward a trajectory leading to a similar/different equilibrium state (recovery) (Lloyd-Price et al. 2016, Ingrisch and Bahn 2018). Thus, the resilience of the microbiota to biotic and abiotic stressors is thought to have important implications for health and welfare, ensuring its ability to withstand disturbances and still maintain its essential functions, structures, and processes.

**Figure 1.**
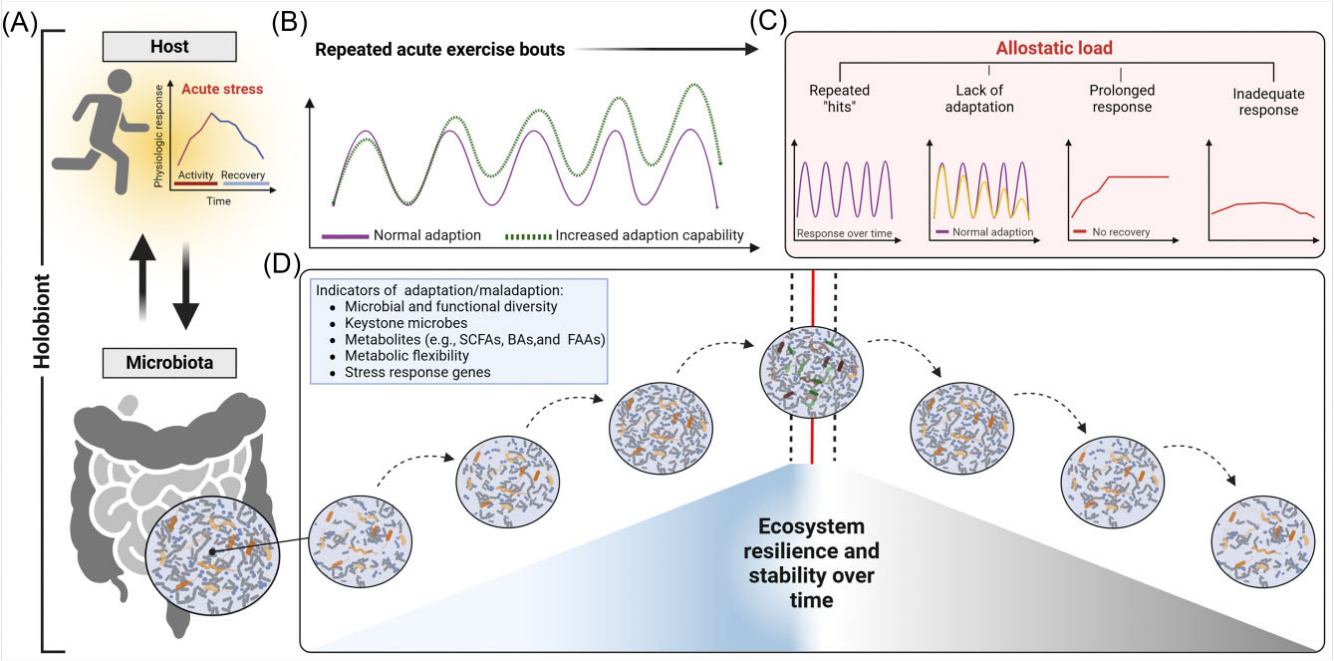
Hypothetical dynamics of exercise-induced GM stability and resilience. (A) Acute exercise acts as a stressor on the host, triggering physiological responses that feed back to and are influenced by the GM. (B) Repeated exercise “hits” can follow a normal adaptation trajectory or an enhanced adaptation capacity. (C) The concept of allostatic load is shown, including maladaptive scenarios such as lack of adaptation, prolonged stress responses, or inadequate recovery. (D) The longitudinal impact of consistent exercise-induced stressors may cumulatively foster significant microbial community shifts, acting as tipping points that trigger transitions to new stable states. These states likely have variable degrees of stability and maintenance of homeostasis, based on the acute ecological pressures for microbial survival (i.e. allostasis). Overtime, conditioned by consistent exercise stress (eustress), the GM may adapt by forming increased resilience, in concordance with a normal allostatic response, and enhancing overall health and performance. Such shifts may be amenable to identifying specific microbial taxa or community structures, which may act as indicators or catalysts of these adaptations. In addition, understanding GM trajectories that may not be optimal, based on different allostatic loads (adapted from McEwen 2000), is also an important consideration in contextualizing stability, resilience, and potential maladaptation. Created with BioRender.com. Abbreviations: SCFAs, short-chain fatty acids; BAs, bile acids; and FAAs, fatty acid amides.

Recently, physical activity has emerged as an abiotic hormetic stressor with potential to shape the GM. Significant physiological changes occur within and around the GI tract during a single bout of physical activity. For instance, prolonged exercise at ≥60% maximum oxygen consumption rate (V̇O_2_ max) redistributes blood flow, resulting in gut hypoxia and hypoperfusion, with reperfusion after the exercise (Costa et al. 2017). These acute perturbations modify the luminal milieu by changing transit rate (Strid et al. 2011), temperature (Yeh et al. 2013), redox balance (Yoon et al. 2024), and—critically—epithelial permeability (Chantler et al. 2021) , thereby permitting the translocation of microbial products and host‐derived metabolites into the circulation (Keirns et al. 2020). Repeated exposure to these effects, akin to the allostatic load theory (McEwen 2000), suggests that the host adapts to such stress through physiological changes that promote resilience. Over time, this may result in the GM evolving into a state that supports the demands of frequent physical activity, aligning with allostasis, where continuous stress leads to adaptation and potentially a new equilibrium in the host’s systemic and microbial environments (Fig. 1). Therefore, similar to the concept of “training the gut” (Jeukendrup 2017), the hormetic stress of exercise that promotes physiological adaptation within the host (i.e. the GI-tract), a parallel and reciprocal process may occur within the GM, aiding in the formation of a distinctive and functional athletic microbiota and a level of ecological resilience (Mohr et al. 2020).

Considerable differences in GM composition and function between athletes and sedentary individuals have been uncovered (Clarke et al. 2014, Barton et al. 2018, Fontana et al. 2023). Thus, the combination of exercise and coinciding behaviors likely influences the gut microbial ecosystem of athletes and other trained individuals. In turn, the GM appears to play roles in fuel availability, muscle function, motivation, overall health, and exercise performance in animal (Dohnalová et al. 2022) and small-scale human research (Clarke et al. 2014, Barton et al. 2018). Notably, germ-free and antibiotic-treated models show that a fully intact GM is crucial for effective muscle adaptation and endurance capacity (Lahiri et al. 2019, Nay et al. 2019). In these studies, removing or disrupting gut microbes reduces running capacity, alters muscle protein synthesis, and impairs the regulation of key metabolic signaling pathways (Lahiri et al. 2019, Valentino et al. 2021, Uchida et al. 2023). Moreover, gut-derived metabolites such as bile acids and neuroendocrine molecules can influence muscle function and motivation (Morville et al. 2018, Sasaki et al. 2018, Qiu et al. 2021, Dohnalová et al. 2022). Exploring how structured physical activity affects the GM and how this microbial community can be influenced by specific inputs or utilized for various health benefits has therefore become a significant focus for exercise researchers, clinicians, trainers, and practitioners (Clark and Mach 2016, Jäger et al. 2019, Mohr et al. 2020, 2022b, a, O’Brien et al. 2022). How exercise stress might, through effects on the GM, potentially accumulate to influence health, welfare, and athletic performance over time, and what happens if this ecosystem goes awry has not been reviewed in detail. In this review, we discuss the features of an exercise-associated GM and synthesize evidence on how exercise and related factors impact GM composition, function, plasticity, and resilience. In addition, we emphasize endurance exercise, largely based on the availability of primary research, to elucidate how associated stressors may compound over time and their systemic repercussions in relation to the holobiont, that is, the assemblage of the host and their associated microbes interacting with each other (Simon et al. 2019). Improving understanding of the exercise-related factors that condition these initial microbial shifts may have important implications for improving physical performance and human health, reducing disease risk, and informing new prospects for restoring dysbiotic ecosystems.

## The athletic GM: linking the GM to an exercise-associated state

**Figure 2.**
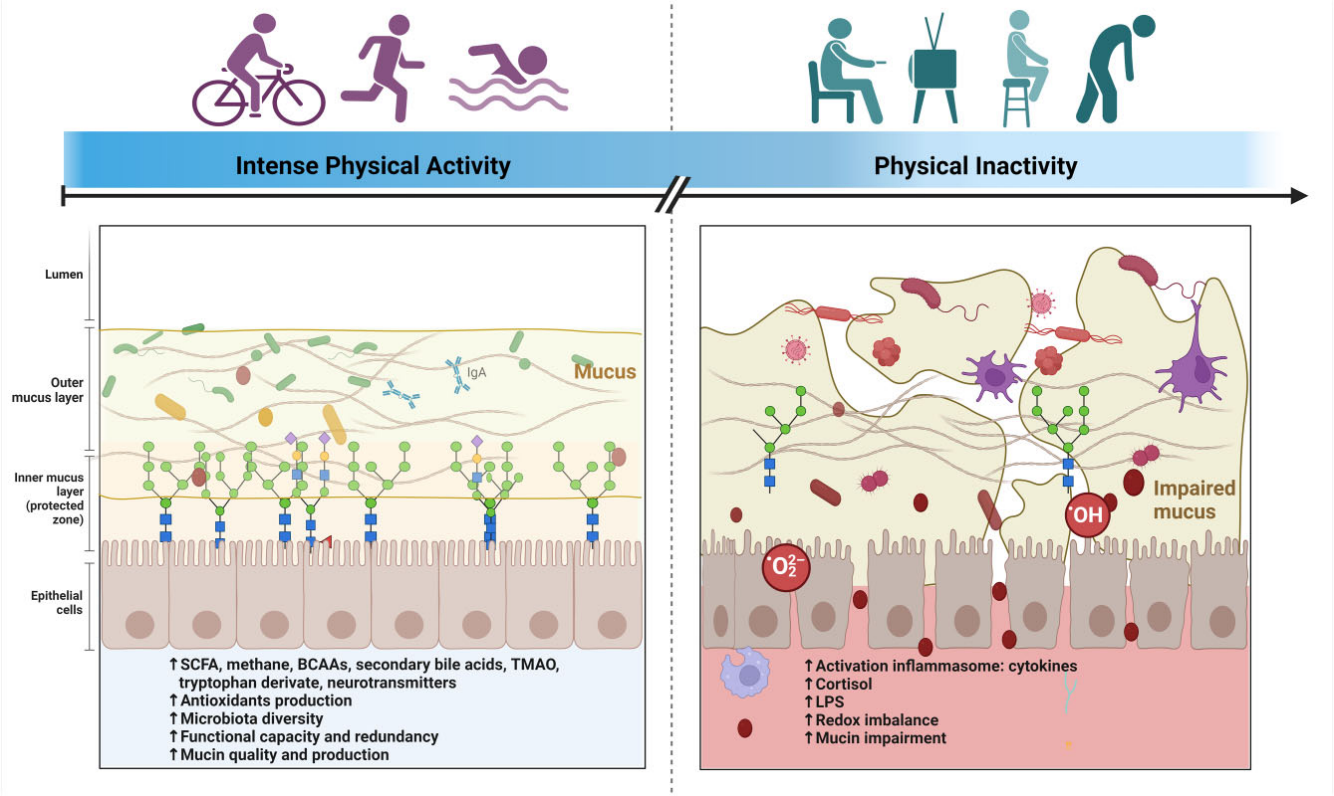
Comparative effects of physical activity versus inactivity on GM and associated host physiology. Conceptual depiction of the beneficial outcomes, such as increased SCFAs, methane, neurotransmitters, and microbiota diversity, alongside improved mucin production and antioxidant levels associated with exercise-associated GM. In contrast, physical inactivity illustrates adverse effects like increased inflammatory cytokines, cortisol, LPS, redox imbalance, and impaired mucin production. These comparisons highlight the potentially profound influence of physical activity on GM and overall host health. Created with BioRender.com. Abbreviations: SCFA, short-chained fatty acids; BCAAs, branched-chain amino acids; TMAO, trimethylamine N-oxide; and LPS, lipopolysaccharide.

Establishing exercise as an influential factor in shaping the GM has largely been driven by observational research attempting to identify GM features and ecological traits characteristic of habitual exercisers compared with sedentary or less active populations (for definitions, see Table 1; Fig. 2). Seminal work in rugby athletes with an overweight body mass index suggested intense physical activity and related factors, such as high-energy/protein dietary intake and circulating markers of muscle damage (i.e. creatine kinase) are associated with greater microbiome richness (Clarke et al. 2014). A metagenomic survey using the same cohort revealed an enrichment of gut mucus-associated bacteria *Akkermansia*, and exercise-related energy pathways and fecal metabolites in the athletes (Clarke et al. 2014, Barton et al. 2018). Similarly, a growing body of research has shown that athletes and regularly active individuals harbor a more diverse GM with enhanced functional capacities than their sedentary counterparts [for in-depth reviews, see Mohr et al. (2020), Cataldi et al. (2022), and O’Brien et al. (2022)]. This is particularly evident in endurance athletes, whose physical activity and dietary patterns may jointly contribute to high microbial diversity and enrichment of specific taxa and unique functions (Petersen et al. 2017, Scheiman et al. 2019, O’Donovan et al. 2020, Li et al. 2023, Shalmon et al. 2024, Humińska-Lisowska et al. 2025).

**Table 1.** Glossary of exercise-related terms relevant to included studies.

Acute exercise	This refers to a single session of structured physical activity. It can vary in duration and intensity, triggering immediate physiological responses like increased heart rate, energy expenditure, and muscle activation. The effects are temporary, subsiding as the body recovers postexercise.
Short-term exercise	This involves repeated sessions of exercise over a shorter period, such as days or weeks (3–4). It can lead to adaptations like improved muscle strength, cardiovascular efficiency, and metabolic changes that are more significant than those from a single session but not as profound or durable as those from long-term exercise.
Long-term (chronic) exercise	This is characterized by regular, ongoing structured physical activity extending over months or years. Chronic exercise leads to long-term physiological adaptations, including increased cardiovascular capacity, muscle hypertrophy, improved insulin sensitivity, and enhanced endurance. These changes are durable and reflect the body’s response to continuous training stimuli.
Exercise intensity	The level of effort required by a particular exercise, categorized as low, moderate, or high. Intensity can be measured through heart rate, oxygen consumption, and perceived exertion.
Exercise mode	The form or type of exercise performed, which influences the specific physiological responses in the body. Modes include aerobic, anaerobic, flexibility, and balance exercises.
Endurance exercise	A type of aerobic activity that involves prolonged physical effort to improve cardiovascular stamina. Common forms include running, cycling, and swimming.
Resistance exercise	Exercise that involves muscle contractions against external resistance with the objective of increasing strength, power, and muscle mass. Examples include weightlifting and resistance bands.
Aerobic exercise	Exercise that use large muscle groups and can be maintained continuously, primarily enhancing cardiovascular fitness by improving the body’s ability to oxygenate blood.
Anaerobic exercise	Intense physical activity that causes lactate to form, typically short bursts of high-intensity movements like sprinting or weight lifting, relying on energy sources within the muscles.
V̇O_2_ max	The maximum volume of oxygen an individual can use during intense exercise, measured in milliliters of oxygen used per minute per kilogram of body weight.
Lactate threshold	The intensity of exercise at which lactate begins to accumulate in the bloodstream, marking the transition from aerobic to anaerobic metabolism.
Functional capacity	The ability to perform tasks and activities efficiently, often improved through exercise tailored to enhance physical strength, endurance, and flexibility.
High-intensity interval training (HIIT)	A training technique involving quick, intense bursts of exercise followed by short, sometimes active, recovery periods. This type of training gets and keeps your heart rate up and burns more fat in less time.
Muscle fiber type 1	Slow-twitch fibers; more efficient at using oxygen to generate fuel for continuous, extended muscle contractions over a long time.
Muscle fiber type 2a	Fast-twitch fibers; a hybrid of type I and type IIb, capable of using both aerobic and anaerobic metabolism.
Muscle fiber type 2b/x	Fast-twitch fibers; excel at producing quick, forceful bursts of speed, and tire out quickly.

Abbreviations: V̇O_2_ max, maximal oxygen consumption.

The rich microbial diversity of the athlete GM encompasses a variety of microbes and functions that may support physical activity. For example, *Akkermansia*, often enriched in athletes (Clarke et al. 2014, Petersen et al. 2017, Martin et al. 2025), promotes gut mucin degradation and regeneration, enhancing energy metabolism, immunity, and barrier function (Ottman et al. 2017). Aerobically trained athletes exhibit higher levels of *Bifidobacterium* (O’Donovan et al. 2020), a genus widely regarded as health-promoting that can degrade host mucins (Ruas-Madiedo et al. 2008, Ruiz et al. 2011, Milani et al. 2015), promote mucus layer growth (Gutierrez et al. 2023), gut barrier function (Abdulqadir et al. 2023), and positively modulate the immune system (Gavzy et al. 2023). Furthermore, *Bacteroides uniformis*, associated with improved exercise performance in both mice and humans, exhibits strong glycolytic capabilities, converting dietary and endogenous glycans into short-chained fatty acids (SCFAs) like acetate and propionate, which can boost hepatic gluconeogenesis and fatty acid oxidation, thereby supplying additional glucose for working muscles (Morita et al. 2023). Additionally, *Methanobrevibacter smithii*, reported to be more abundant in athletes (Petersen et al. 2017), enhances methane production and energy and carbohydrate metabolism, potentially increasing energy availability and reducing recovery times (Clark and Mach 2023). Athletes’ GM also show greater potential for producing compounds like vitamin B12, amino acid derivatives (Fontana et al. 2023), and endocrine metabolites and neurotransmitters (Dohnalová et al. 2022), all of which may promote exercise performance.

Recent research indicates that the GM of athletes may be tailored to their specific discipline and exercise demand, suggesting that exercise patterns (i.e. mode, intensity, and duration) rather than or in addition to exercise alone critically influence the ecosystem (Li et al. 2023). For instance, athletes involved in high-dynamic sports (e.g. marathon and field hockey) exhibit a distinct microbial composition with higher levels of genera such as *Bifidobacterium, Lactobacillus, Prevotella*, and *Faecalibacterium* compared to those in lower-dynamic sports (e.g. judo and taekwondo) (O’Donovan et al. 2020). A recent comprehensive metagenomic study by Fontana et al. (2023) analysed 185 high-level athletes, 69 mid-level athletes, and 166 sedentary individuals, revealing a microbial pattern dominated by SCFA-producing microbes and a significantly higher number of carbohydrate-active enzymes in athletes. Collectively, these studies demonstrate that the GM of athletes and other trained individuals is more taxonomically and functionally diverse than those of less active counterparts, and within athlete populations, differences in exercise patterns and demands may further drive ecological differences.

Different athletic disciplines can also dictate different dietary patterns, which in turn affect the GM. Aerobic athletes typically consume more carbohydrates and fiber (Vitale and Getzin 2019) compared to anaerobic athletes, who often have higher protein and fat intakes (Antonio et al. 2015). Such dietary differences can impact the GM, as evidenced in a comparison of aerobic and anaerobic athletes, where significant differences in protein and fiber intake potentially confounded microbiome results (Jang et al. 2019). Similarly, a longitudinal study of rowing athletes showed that despite greater microbial diversity and enhanced metabolic functions in elite rowers, up to 29% of the interindividual variation was attributed to differences in daily nutrient intake (Han et al. 2020), supporting that diet can overshadow exercise effects. The role of dietary fiber is particularly notable, as various fibers differentially influence GI motility, transit time in the colon (Gear et al. 1981), mucus layer thickness and nutrient cycling (Desai et al. 2016), factors that are crucial in shaping the GM’s diversity, genomic regulation, and intra- and interkingdom communication (Roager et al. 2016, Asnicar et al. 2021, Steenackers et al. 2022). Additionally important is colonic pH. Human colonic pH spans roughly 5.0–7.5 and, independent of transit time, is now recognized as a key selector of microbial ecology; mildly acidic conditions (<6.5) favor saccharolytic, butyrate-producing taxa, whereas neutral or alkaline pH shifts metabolism toward proteolytic pathways, branching-chain fatty acids, and other alkaline metabolites (Brinck et al. 2025). Because fecal pH is remarkably stable within individuals yet varies widely between them, pH differences can explain a measurable fraction of interindividual variation in both microbiota and metabolome profiles (Brinck et al. 2025). Exercise itself has been found to accelerate GI transit time (Keeling and Martin 1987, Jensen et al. 2023), contrasting with sedentary behavior, which is often linked with constipation (Huang et al. 2014). Additionally, metabolites commonly produced in an athlete-associated GM, such as butyrate (Haschke et al. 2002), lithocholic acid (Li et al. 2021), and histamine (Chen et al. 2019), can stimulate gut motility, affecting transit time. As such, disentangling the specific contributions of exercise and diet to the GM is challenging due to overlapping effects and the multifactorial nature of these influences.

Considered together, cross-sectional findings of studies indicate that athletes and physically active individuals exhibit distinct fecal microbiota profiles compared to sedentary populations. However, the observational nature of most studies and potential confounding (such as diet) leave open questions of causality. Further, interpreting fecal bacterial profiles as definitive indicators of gut health requires caution since microbial communities vary considerably along the GI tract and may not be fully represented in stool samples. Many studies profiling athletes have also relied on 16S rRNA sequencing (Clarke et al. 2014, Jang et al. 2019, Kulecka et al. 2020), which offers less resolution than whole metagenomic/transcriptomic analyses, further limiting the granularity of these observations. Therefore, while provocative, these studies cannot answer the question of whether exercise itself drives GM differences between physically active versus more sedentary population, or whether observed differences are primarily related to diet, initial GM composition, or other phenotypic or physiologic attributes. However, as we discuss below, it is biologically plausible for exercise to be an important driver of GM composition and function based on physiologic and metabolic responses to exercise that may cumulatively, over time, shape the GM ecosystem.

## Hormetic stressors influencing acute effects of exercise on the GM: the endurance model

**Figure 3.**
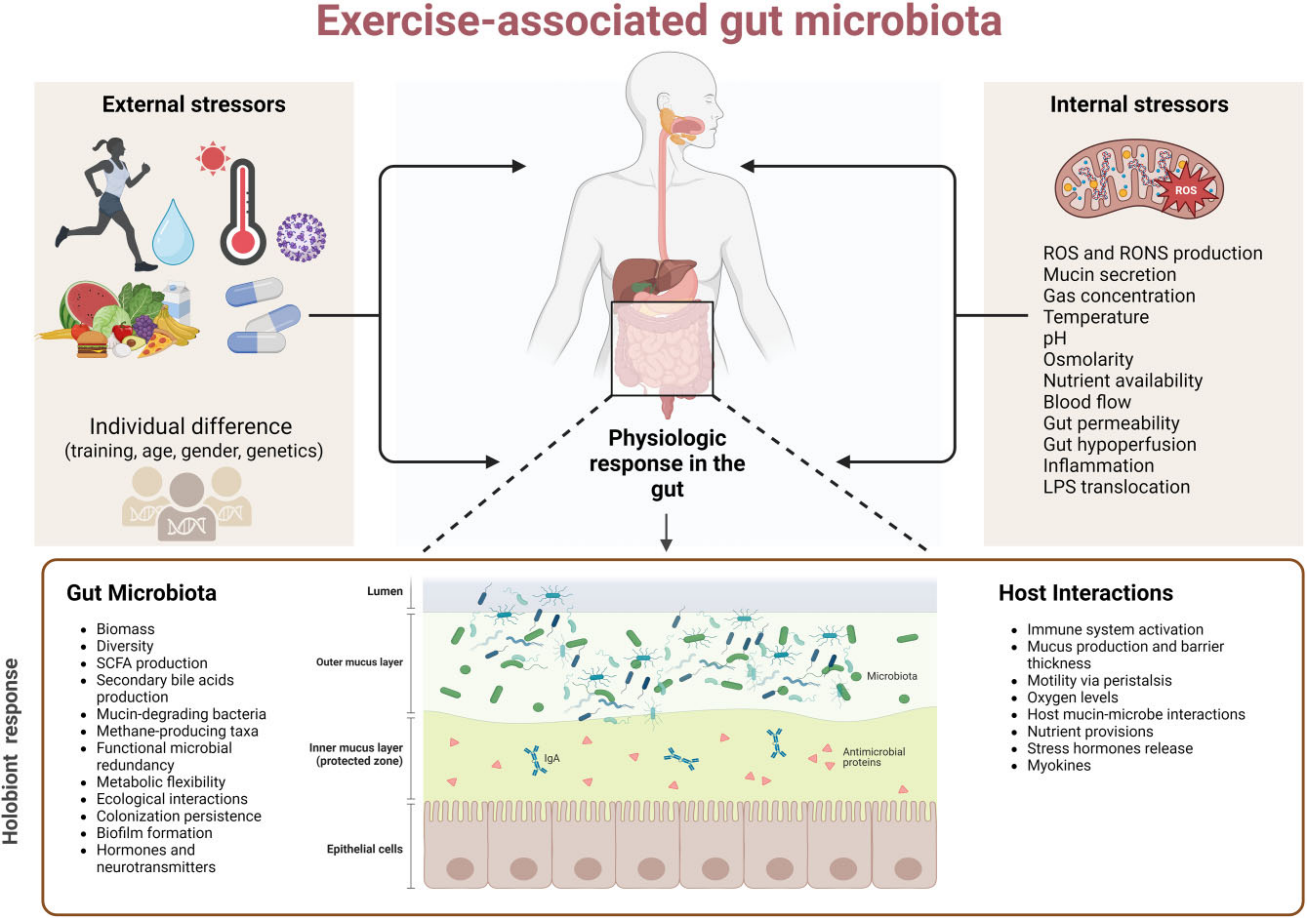
The hormetic effects of exercise on GM dynamics. Internal and external factors collectively orchestrate acute modifications in the GI tract that impact the GM. Internally, factors such as mitochondria function, mucin secretion, gas levels, pH balance, blood flow redistribution leading to intestinal ischemia, hydration status, redox balance, nutrient access, and inflammation may play a role. Externally, diet, thermic stress, medications, and the intensity and duration of exercise all could contribute to the acute modulation of the GI tract and GM. Overall, the immediate effects of endurance exercise on the GM encompass a broad spectrum of mechanical, thermal, biochemical, and physiological shifts. These interactions underline the nuanced response of the GM to varying degrees and types of physical stress, pointing to the importance of tailored exercise regimens for optimal gut health and microbial resilience. Created with BioRender.com.

Endurance exercise acts as a hormetic stimulus for the GI tract. A single bout of moderate- or high-intensity work can transiently reduce splanchnic blood flow, raise luminal temperature, and loosen epithelial tight junctions, producing a short-lived rise in gut permeability (Marchbank et al. 2011, Zuhl et al. 2014, Keirns et al. 2020). When the same stimulus is applied regularly and progressively, the gut adapts; barrier function improves, GI-disease risk falls, and overall health markers rise (Peters et al. 2001). This creates the familiar “J-shaped” relationship in which both inactivity and excessive training load increase GI vulnerability, whereas moderate, periodized exercise is protective (O’Brien et al. 2022). In practice, however, many endurance athletes still report GI symptoms during or after intense sessions (Oliveira et al. 2014, Pugh et al. 2018). As described below, intense physical activity exposes the gut to several acute stressors—mechanical, thermal, and physiological—instigating a holistic holobiont response (Fig. 3) that varies according to factors like modality, intensity, and duration of exercise. Differences in these factors can elicit varied protective or detrimental effects on gut health and metabolic processes (e.g. substrate utilization, SCFA production, and energy homeostasis), thereby providing an ideal framework for studying short-term exercise–GM interactions that cumulatively shape long-term GM adaptability, stability, and resilience.

### Redistribution of blood flow, immune activation, and gut barrier function

During exercise, blood flow is significantly redirected from the splanchnic circulation to working muscles and skin, reducing splanchnic blood flow by up to 50% (Qamar and Read 1987, Osada et al. 1999). This can cause gut ischemia and reperfusion that together increase brush border permeability (Wijck et al. 2012) and cause gut barrier damage via increases in oxidative stress and inflammation (Otte et al. 2001). High-intensity or prolonged exercise (i.e. ≥70% V̇O₂ max), in particular, often increases gut permeability, especially when conducted in hot environments (Marchbank et al. 2011, Zuhl et al. 2014), allowing luminal components to leak into circulation (Wijck et al. 2012, Costa et al. 2017), where they stimulate an immune response and subsequent inflammation (Shing et al. 2014, Zuhl et al. 2015).

Changes within the GI environment resulting from oxidative stress, inflammation, and gut barrier damage may influence the GM, possibly promoting GM adaptations that may improve barrier function over time. In support, ischemia/reperfusion can promote increases in anti-inflammatory taxa such as *Bacteroides vulgatus* in animal models (Deng et al. 2021). *Bacteroides vulgatus* gavage in mice decreases gut microbial lipopolysaccharide (LPS) production, suppressing proinflammatory immune responses (Yoshida et al. 2018) and attenuating symptoms of dextran sodium sulfate-induced colitis (Liu et al. 2022a). Further, specific strains of *B. vulgatus* (SNUG 40005) decreased gut permeability and increased the abundance of *Akkermansia muciniphila* in mice fed a high-fat diet (You et al. 2023). That response may reflect a cross-feeding interaction as *Bacteroides* act on dietary carbohydrates and supply other colonic bacteria with various digested glycan sources, creating a biocompatible GM environment (Sonnenburg et al. 2016). Indeed, providing nutrients to *A. muciniphila* can indirectly improve gut barrier integrity by supporting the mucus layer (Everard et al. 2013), upregulating tight junction proteins (Chelakkot et al. 2018), and increasing antiinflammatory Treg cells (Shin et al. 2014).

Ultimately, GM responses caused by changes in the GI environment and resulting interactions within the community may comprise hormetic effects that enhance barrier function over time (Keirns et al. 2020). In support, exercise regimens as brief as 2 weeks can positively affect epithelial cell and mucosal turnover (Motiani et al. 2020), enhancing gut barrier resilience and stability (Pasini et al. 2019). Trained athletes often exhibit lower baseline levels of gut permeability markers, such as LPS, than sedentary individuals (Lira et al. 2010). This suggests that regular exercise fortifies barrier functions, possibly by enhancing tight junction integrity, mucin production and quality, mucosal immune responses, and anti-inflammatory and antioxidant defenses (Hoffman-Goetz et al. 2010, Packer and Hoffman-Goetz 2012, Allen et al. 2018a). Improved barrier health may also be facilitated by promoting beneficial taxa within the GM, as described above, in addition to shifting the GM towards butyrate-producing communities enriched with taxa including *Lachnospiraceae* and *Faecalibacterium prausnitzii* (Matsumoto et al. 2014, Campbell et al. 2016), that stimulate the production of butyrate and other beneficial metabolites (Wang et al. 2012, Yan and Ajuwon 2017). Increasing butyrate in the gut can enhance barrier integrity and exert systemic anti-inflammatory effects, partly by inducing intestinal T regulatory cells that produce anti-inflammatory cytokines like IL-10 (Furusawa et al. 2013, Lorén et al. 2015). Thus, acute exercise reduces splanchnic blood flow leading to oxidative stress, inflammation, and temporary increases in gut permeability. In contrast, although many mechanistic details are unclear, chronic exercise may foster a resilient gut barrier and complementary shifts in the GM that reinforce those effects.

### Thermal stress and hydration

During strenuous endurance sports, core body temperature can rise from a resting range of 36.1°C–37.2°C (Hoffmann et al. 2012, Huus and Ley 2021) to roughly 40°C, depending on environmental conditions (Noonan et al. 2012). Elevated core body temperature causes blood to be shunted toward the body surface to maximize heat loss, resulting in vasoconstriction in the GI tract (Lambert 2009), ischemia, and increased gut permeability (Pires et al. 2017). These physiologic responses to thermal stress reduce nutrient and oxygen delivery to the intestinal lumen and alter local immune signaling, which may contribute to exercise-induced shifts in the GM.

Though research on the GM of heat stressed athletes is limited, one study of endurance-trained athletes running at 60% V̇O₂ max in ∼35°C conditions reported modest negative correlations between core body temperature and *Prevotellaceae* and a positive correlation between core body temperature and *Ruminococcaceae* (Bennett et al. 2020). In animal studies, heat-stressed livestock experienced decreases in alpha diversity and Bacillota abundance (Tajima et al. 2007, Zhu et al. 2019, Wang et al. 2020) and increases in relative abundances of Pseudomonadota (Sciellour et al. 2019). Rats exposed to constant temperatures of 35°C–38°C for 7 days exhibited significant reductions in *Lactobacillus* and *Bacteroides*, while *Oscillospira* and *Clostridium* levels rose (Qu et al. 2021). In that study, shifts in GM community function were also noted, and characterized by increased expression of genes related to carbohydrate and amino acid metabolism, and membrane transport. By contrast, when rats were subjected to intermittent heat stress (35 ± 1°C and 60 ± 5% humidity for 120 min/day over 28 days), alpha diversity and *Lactobacillus* relative abundance increased (Cao et al. 2022). This rise in *Lactobacillus* differs from the decrease observed under continuous heat exposure (Qu et al. 2021), suggesting that exposure pattern (intermittent versus continuous) and duration can yield distinct microbial outcomes. Notably, *Lactobacillus* is known for its role in immune defense and protecting epithelial cells, which may protect gut barrier function (Gareau et al. 2010).

In controlled settings, high ambient and core body temperatures can lead to underhydration. In mice, underhydration diminishes colonic immune cell function and pathogen elimination (Sato et al. 2024). Hydration also impacts intraluminal osmotic pressure, stool consistency, and GM composition (Useros et al. 2015). Notably, individuals with low compared to high water intake have different microbial community structures and greater abundances of *Campylobacter* (Vanhaecke et al. 2021), a genus associated with GI infections (Igwaran and Okoh 2019). Thus, both hydration and body temperature are likely crucial factors influencing the hormetic effect of exercise on the GM.

### GI transit time

In healthy individuals, transit times vary widely (Procházková et al. 2022), influenced by factors such as diet, body weight, height, and sex (Degen and Phillips 1996, Nandhra et al. 2020). Intestinal transit rate is also influenced by acute endurance exercise (Horner et al. 2015), affecting the microbial environment due to changes in pH and nutrient availability in the colonic lumen. While gastric emptying appears to be delayed at higher exercise intensities (i.e. above 70% V̇O_2_ max) (Feldman and Nixon 1982, Horner et al. 2015), habitual physical activity, particularly aerobic exercise, enhances GI motility (Bingham and Cummings 1989), thereby reducing transit times and potentially improving irregular bowel habits (Tottey et al. 2017). These variations in transit time alter substrate availability within the colon, which, in turn, impacts microbial growth dynamics, colonic pH, and stool output even under a constant diet (Stephen et al. 1987, Asnicar et al. 2021). Specifically, slow colonic transit reduces carbohydrate availability in the distal colon (Procházková et al. 2022), favoring bacteria that utilize alternative energy sources, such as dietary or host-derived proteins (Roager et al. 2016). Resultantly, extended transit times have been associated with increased microbial diversity, *Akkermansia* and *Methanobrevibacter* abundance, pH levels, and GM-derived metabolites associated with proteolytic metabolism (Tottey et al. 2017). In contrast, faster transit times are associated with increased capacity for saccahrolysis and SCFA production (Procházková et al. 2022). Thus, the interplay between physical activity and gut transit time may be an additional factor influencing the effects of exercise on GM stability and resilience.

### Coordinated metabolic response to exercise: connecting the GM to host tissues

Beyond the gut, acute exercise orchestrates a systemic adaptative change continuum within the host (Gabriel and Zierath 2017). This continuum influences host protein turnover, glucose uptake, fat utilization, and skeletal muscle metabolism to maintain the required rates of adenosine triphosphate (ATP) synthesis while minimizing disturbances to cellular homeostasis. The GM contributes to the host’s metabolic landscape, producing or altering a substantial portion of gut metabolites (>830 gut microbial metabolites identified in humans) (Zheng et al. 2011). These metabolites represent a vast pool of bioactive compounds crucial for cross-feeding activities between microorganisms (Han et al. 2021). However, GM-derived metabolites also influence host physiology beyond the gut including within the brain and muscle, which may link exercise-associated change in the GM to host adaptations to exercise (Chow et al. 2022). Accordingly, a holo-omic approach, which incorporates multiomic data from both the host and microbiota domains, is increasingly being implemented to untangle the interplay between the two.

Pivotal studies have demonstrated that transferring the microbiome of exercise-trained donors into germ-free mice enhances key metabolic pathways in muscle tissues, such as AMP-activated protein kinase, calcium/calmodulin-dependent protein kinase II, and Akt substrate of 160 kDa (Aoi et al. 2023). An intact GM is required for exercise‐induced muscle adaptations, including endurance exercise capacity (Okamoto et al. 2019) and skeletal muscle hypertrophy (Valentino et al. 2021). Some of the most prominent signaling agents contributing to microbiota–gut–muscle communication are SCFAs. A significant fraction of colon-derived acetate, and to a lesser extent propionate and butyrate, reaches systemic circulation and peripheral tissues, such as the muscle and brain (Dalile et al. 2019), potentially influencing the physiological and psychological functioning of the athlete via immune, endocrine, neural, and humoral routes. For example, in exercise-naïve mice, SCFA administration improved oxidative capacity and mitochondrial function (Ismaeel et al. 2023). Other SCFAs, such as butyrate, inhibit glycolysis and switch cell metabolism toward gluconeogenic conditions, thus promoting lactate utilization (Morand et al. 1994). Butyrate can also shift muscle fibers toward a more oxidative phenotype by inhibiting histone deacetylases and promoting PGC-1α-driven mitochondrial biogenesis (Gao et al. 2009).

Emerging evidence shows that cross-talk in the gut–muscle axis may also occur through communication between GM-derived metabolites and the mitochondria. Mitochondria, which are highly responsive to microbiota signaling, share maternal inheritance patterns with the GM, revealing a close evolutionary relationship, particularly evident in cytochrome lineage (Andersson et al. 1998). Traditionally recognized for their role in bioenergetics through pathways like oxidative phosphorylation and fatty acid β-oxidation, mitochondria are now recognized for a broader range of functions. Those functions include regulating cytosolic calcium, maintaining cellular redox status, generating reactive oxygen species (ROS), and participating in processes such as steroid and heme biosynthesis, apoptosis, and inflammation initiation (Jackson and Theiss 2020).

The complex interplay between GM and the mitochondria occurs principally through endocrine, immune, and humoral signaling (Mottawea et al. 2016). Microbial metabolites, including SCFAs, branched-chain amino acids, and secondary bile acids, are all implicated in mitochondrial biogenesis and function (Andersson et al. 1998, Clark and Mach 2017). Moreover, variations in the mitochondrial genome are correlated with GM diversity, linking reduced microbial diversity to increased ROS production (Yardeni et al. 2019). Evidence highlights the interdependence of mitochondria and GM, particularly during exercise. For example, Mach et al. (2021b) demonstrated that endurance athletes harbor functionally redundant butyrate-producing bacteria, such as those from the *Lachnospiraceae* family, which are associated with mitochondrial gene regulation. Effects of butyrate on the mitochondria include promoting the expression of PPARγ, facilitating fatty acid oxidation (Gao et al. 2009), and possibly maintaining redox balance during strenuous exercise (Mottawea et al. 2016).

Beyond SCFAs, secondary bile acids and H_2_ are essential for mitochondrial–GM cross-talk. Secondary bile acids may interact with mitochondria via the FXR-CREB axis (Seok et al. 2014), while H₂ regulates mitochondrial function through the *PPARγ*/*Pgc-1α*/*Tfam* pathway. Research by Luo et al. (2022) demonstrated that H₂-rich water enhances exercise tolerance in rats by promoting GM diversity and upregulating mitochondrial biogenesis. Thus, under conditions that enhance the colonization of a complex functional microbiome capable of producing beneficial metabolites, mitochondrial functionality and athletic performance may be positively influenced.

Investigations by Yardeni et al. (2019) revealed that the GM could be modulated by mitochondrial redox status and ROS production. They found significant differences in NAD+/NADH ratios between mice with mtDNA variants associated with higher ROS production and control mice. Since the NAD+/NADH ratio is critical for regulating mitochondrial metabolism, such changes could influence gut microbial populations. The intermittent increase in ROS during exercise may foster adaptive mechanisms within the GM, altering its composition and functionality. While mechanisms underpinning direct effects of redox status on the GM are not fully clear, exercise intermittently increases ROS. Such pulses on the GM may promote adaptive and protective mechanisms, thus altering the community composition and function.

Finally, within the context of coordinated responses to exercise-associated factors (i.e. thermal stress, GI transit, ROS production, metabolic milieu, and so on) microbial communities also adapt by forming protective structures such as microcolonies and biofilms in the gut. This response enhances resilience to the fluctuating gut environment. Microcolonies are small aggregates of bacteria and form under suboptimal conditions, providing a fitness advantage over nonaggregated counterparts (Burmølle et al. 2014). These formations represent some of the simplest multicellular assemblies and can establish strong presences in small niches within the gut (Tytgat et al. 2019). Biofilms, consisting of mixed microbial communities encased within a matrix, adhere to biotic and abiotic surfaces and facilitate nutrient exchange, stress resistance, and cross-feeding, establishing a robust digestive consortium (Hooper and Gordon 2001, Stoodley et al. 2002, Otto 2014). Repeated strenuous exercise has been shown to stimulate microcolony and potentially biofilm formation in sheltered areas of the gut like the appendix and crypts (Stoodley et al. 2002). Exploring microcolonies and biofilms within GM in the context of physical activity is a relatively uncharted area of research that holds potential for novel insights.

## Plasticity of GM: short-term responses to exercise

Over time, the exercise-induced physiologic and metabolic responses described above, along with accompanying factors like dietary intake, can induce measurable changes in GM structure, function, and dynamics (Mohr et al. 2020). In line with the concept of hormesis, those changes can potentially foster a health-associated state within the host wherein “healthy” microbial communities maintain self-regulation in the long term. Conversely, when training loads are excessive or recovery is insufficient, the cumulative stress can temporarily destabilize the ecological state of the GM and potentially impair host health (Karl et al. 2017, Pugh et al. 2017, Craven et al. 2022, Lian et al. 2024). Thus, the GM’s response to acute and shorter-term (∼3–4 weeks) exercise-induced perturbations offers insight into the resilience mechanisms of individual microbial ecosystems that ultimately are pivotal in establishing a foundation for long-term physiological (Dawson et al. 2018) and potential GM adaptations (Grosicki et al. 2023a). Below we discuss evidence from both animal and human studies that demonstrate how exercise-induced physiologic and metabolic responses manifest within the GM ecosystem.

### Animal models: exercise-induced impacts on the GM

Research in rodent models has generally shown that exercise can significantly alter the GM’s composition and function. These changes are evident within weeks and vary depending on the duration and type of exercise (Choi et al. 2013, Queipo-Ortuño et al. 2013, Evans et al. 2014, Allen et al. 2015, Denou et al. 2016, Carbajo-Pescador et al. 2019, Fernández et al. 2021, Yang et al. 2021, Imdad et al. 2024). For example, several studies have reported increased abundance of *Lactobacillus, Bifidobacterium* (Queipo-Ortuño et al. 2013), and *Turibacter* spp. (Allen et al. 2015), alpha diversity (Choi et al. 2013, Denou et al. 2016, Imdad et al. 2024), and cecal butyrate (Matsumoto et al. 2014) following exercise intervention. However, other studies have reported no significant changes in overall diversity and GM structure with exercise alone (Zhang et al. 2013, Lamoureux et al. 2017, Brandt et al. 2018, Ribeiro et al. 2019). Those inconsistencies highlight a need for further research on factors influencing effects of exercise on the animal GM include different exercise patterns, dietary conditions, host characteristics (e.g. sex, obese status, and so on), forced versus voluntary exercise, and analytical techniques (e.g. GI region, sequencing method, and so on).

Results from studies using larger mammals like horses are similarly inconsistent. For example, participation in a 1900 m race significantly altered levels of certain microbial taxa like Bacillota and Bacteroidota in Thoroughbred racehorses (Górniak et al. 2021). However, intense acute exercise or prolonged training showed no effect on the fecal microbiome of Standardbred racehorses (Janabi et al. 2016). Other work suggests endurance horses maintain a stable microbiota composition after racing, though their metabolic functions show significant shifts, as indicated by changes in plasma metabolites (Mach et al. 2021b). Mach et al. (2022) also mapped the metagene catalog of the baseline GM in elite endurance horses to better understand any role in fatigue resistance and performance enhancement. Their findings highlighted a negative correlation between the presence of *Lachnospiraceae* taxa and cardiovascular capacity, whereas more complex and functionally diverse microbiomes were linked to higher glucose concentrations and lower levels of long-chain acylcarnitines and nonesterified fatty acids in plasma. Interestingly, methane-producing taxa and mucin-degraders were more prevalent in the GM of fitter animals. While these animals cannot absorb methane (Shabat et al. 2016), it may exert anti-inflammatory, antiapoptotic, and antioxidative effects in the gut (Boros et al. 2012). This suggests that methane-producing taxa, typically associated with energy waste, might contribute to protecting the gut from the inflammatory effects of endurance exercise. Additionally, the higher intestinal abundance of Verrucomicrobiota (e.g. *A. muciniphila*) and the enrichment of carbohydrate enzymes able to cleave host mucin glycans illustrated the possibility that the makeup of the GM and their functional capacity in racehorses are primed for gut mucosa repair (Mach et al. 2022). Such characteristics imply that repeated exercise stress could select for microbial traits that enhance gut barrier repair and resilience, wherein successive exposures prime the microbial community to better withstand similar future stresses (i.e. ecological memory).

As summarized in Table 2, these and other findings support the interplay of exercise, diet, and host factors in shaping the structure and function of the GM in animal models, as well as the potential for certain microbial taxa or metabolic profiles to confer resilience and performance benefits.

**Table 2.** Thematic overview of exercise-induced GM changes in animal models.

Theme	Key observations	Representative supporting evidence
Exercise in rodents	• Even brief exercise (6 days–5 weeks) can modulate GM composition and function • Longer exercise regimens (≥12 weeks) more consistently increase alpha-diversity and beneficial taxa • Some rodent studies show no significant changes in overall diversity with exercise alone, highlighting interstudy variability	• 6 days of exercise modulated *Lactobacillus* and *Bifidobacterium* in rats (Queipo-Ortuño et al. 2013) • 5 weeks of voluntary exercise shifted GM abundance and composition (Choi et al. 2013) • 12 weeks increased alpha-diversity, improved bacteroidota/bacillota ratio (Imdad et al. 2024) • No major GM changes in some studies (Zhang et al. 2013, Lamoureux et al. 2017, Brandt et al. 2018, Ribeiro et al. 2019)
Equine models: species-specific responses	• Horses, especially endurance breeds, often exhibit GM resilience, with minimal compositional changes pre- versus postrace • Metabolic activity shifts significantly, as seen in plasma metabolite profiles • Thoroughbreds versus standardbred responses can differ, highlighting individual variation	• Endurance horses show stable GM composition but altered metabolite profiles (Mach et al. 2021b) • Participation in a 1900 km race ↑ Bacillota and Bacteroidota in thoroughbreds (Górniak et al. 2021) • No significant changes in standardbred faecal microbiome after acute intense exercise or 12 weeks of training (Janabi et al. 2016)
Exercise–diet interactions	• Exercise partially counteracts high-fat diet-induced dysbiosis • Maintains intestinal barrier function, improves bile acid homeostasis, and modulates metabolic profiles under dietary restriction • Restricted feeding plus exercise alters bile salt hydrolase-expressing microbes	• Exercise preserved GM composition versus high-fat diet imbalance (Carbajo-Pescador et al. 2019) • 6 days of exercise + restricted diet altered *Lactobacillus* and *Bifidobacterium* (Queipo-Ortuño et al. 2013) • High-intensity interval training restored Bacteroidota/Bacillota ratio in mice on a high-fat diet (Denou et al. 2016)
Butyrate production	• Exercise increases the abundance of butyrate-producing microbes • Butyrate improves gut integrity, supports mucin synthesis, decreases epithelial permeability, and reduces serum ghrelin	• 12 weeks of voluntary exercise ↑ butyrate-producers (Evans et al. 2014) • Butyrate’s effects on gut barrier and mucin (Burger-van Paassen et al. 2009, Lewis et al. 2010, Peng et al. 2009) • Lower ghrelin levels with butyrate (Lin et al. 2012)
Resilience	• A higher baseline microbial diversity and functional redundancy may foster GM resilience under physical and emotional stress • Metrics beyond alpha-diversity (e.g. metagenetics, gene flow, and functional capacity) could better capture ecosystem stability • Microbiota-targeted interventions could modulate exercise outcomes, but more research is needed	• 14 weeks of continuous exercise altered core bacterial activity (Yang et al. 2021) • High diversity linked to better metabolic health, performance, and lower levels of inflammatory markers (Mach et al. 2022) • Recommendations for next-generation resilience metrics (Mach et al. 2022)

### Human research: exercise and GM dynamics

Research on acute and short-term endurance exercise in human athletes has revealed dynamic GM changes. For instance, a study of amateur runners demonstrated rapid shifts in the fecal metabolome after a half-marathon, including an expansion of *Coriobacteriaceae* involved in bile salt and steroid metabolism (Zhao et al. 2018). Conversely, the pentose phosphate pathway, crucial for nucleotide synthesis (Alfarouk et al. 2020), and pathways for essential amino acids like phenylalanine, tyrosine, and tryptophan were reduced, aligning with the energy demands of prolonged physical activity and possibly influencing mood and fatigue (Strasser et al. 2016)). A common finding reported from pre- to post-event in marathon and ultramarathon runners has been an increase in *Veillonella* species (Grosicki et al. 2019, Scheiman et al. 2019), known for converting lactate into SCFAs such as acetate and propionate via the methylmalonyl–CoA pathway (Ng and Hamilton 1973). Notably, *V. atypica* supplementation has been shown to preserve exercise performance during strenuous activity (Gross et al. 2023). Other acute exercise responsive taxa, such as *B. uniformis*, can facilitate hepatic gluconeogenesis, supporting the theory that microbial adaptations to strenuous exercise may bolster endurance (Morita et al. 2023). Collectively, these findings reinforce the “form fits function” hypothesis wherein host metabolites produced during exercise, such as lactate, may serve as substrates for specific bacterial taxa who transform those compounds into other metabolites that may benefit the host.

Methanobacteria, the archaeal inhabitants of the gut, appear to be a cornerstone for understanding the intricate symbiosis between host and microbe during exercise. For example, *M. smithii* has been reported to be the dominant methanogen in the human GM (Dridi et al. 2009) and has been associated with increased community diversity and metagenomic richness (Lahti et al. 2014). As noted in cyclists, *M. smithii* enrichment has been found primarily in the most competitive participants. The organism’s high capacity for methane production has broad implications for other metabolic processes, including those related to SCFA production. By consuming H₂ and CO₂ byproducts of bacterial fermentation, to produce methane *M. smithii* enhances abundances of fermenting bacteria and fermentative efficiency within the GM (Vanderhaeghen et al. 2015) through trophic interactions with fermenters like *B. thetaiotaomicron* (Catlett et al. 2022). Large-scale metagenomic surveys further show that *M. smithii, Prevotella, Ruminococcus*, and *Collinsella* often form a syntrophic guild, facilitating complex polysaccharide degradation (Bai et al. 2022). Additionally, a meta-analysis of metagenomes across populations found that the bacterial family *Christensenellaceae* and the archaeal family *Methanobacteriaceae* often cooccur and are more prevalent in individuals with a lean body mass index (Ruaud et al. 2020), and increase during weight loss (Mohr et al. 2024). Coculturing experiments suggest a syntrophic relationship between *Christensenella* species and *M. smithii*, possibly through interspecies hydrogen transfer (Ruaud et al. 2020). Such interactions favor the production of acetate over butyrate, potentially promoting a lean phenotype. Indeed, under controlled feeding conditions, methane production by methanogens has been reported to contribute to a net negative energy balance (Corbin et al. 2023). Therefore, cooperation among microbes likely aids in balancing hydrogen content in the gut for optimized fermentation of carbohydrates and methane producers may be critical in driving an exercise-associated GM.

Acute shifts in the GM following extreme physical activity can also induce changes perceived as likely less beneficial. For example, a 4-day cross-country ski march in Arctic conditions led to higher alpha-diversity but also increased GI permeability, inflammation, and a rise in less dominant taxa, including potential pathogens, alongside reductions in beneficial groups such as *Bacteroides, Faecalibacterium, Collinsella*, and *Roseburia* (Karl et al. 2017). Moreover, in response to ultramarathon events, significant increases in potentially pathogenic taxa have been noted in a highly fit runner (Grosicki et al. 2019) and an individual with obesity (Saragiotto et al. 2024). These alterations indicate a complex, allostatic response of the GM to the stress of extreme physical exertion, which may influence the incidence of postexercise infections and inflammation. Despite these changes, the highly fit athlete reported no severe GI complaints, which may highlight resilience within the gut system under extreme conditions. At the same time, it raises intriguing questions about the implications of the proliferation of potentially pathogenic bacteria in response to allostatic load from extreme endurance exercise, especially because many marathon and ultramarathon runners experience acute deterioration and inflammation of the GI mucosal barrier during or after exercise.

At the opposite end of the spectrum, physical inactivity presents an opportunity to explore how the GM adapts and responds to different allostatic loads. In humans undergoing severe hypoactivity (via dry immersion, a method to simulate the effects of weightlessness by immersing a subject in a specially designed tank or bed filled with water), muscle atrophy occurred alongside changes in the GM, including increased *Clostridiales* and *Lachnospiraceae*, anaerobic glycolysis pathway impairment, and decreased fecal propionate (Jollet et al. 2021). As previously mentioned, *Lachnospiraceae* taxa correlated negatively with cardiovascular capacity in an animal model and occupied niches of noncore taxa that contributed to performance (Mach et al. 2022). Thus, hypoactivity may drive taxonomic and functional changes in the GM that potentially impair host metabolic capacity, contrasting sharply with the adaptive benefits often seen in physically active populations

While the overall composition of the GM often remains resilient to acute bouts of high physical demand, the community’s metabolic activity is much more responsive. In a study assessing the GM in response to an Ironman triathlon, despite stability in microbial composition, significant changes were noted in the fecal metabolome, particularly in bile and fatty acid profiles (Grosicki et al. 2023b), which parallels findings observed during a 4-day cross-country ski march (Karl et al. 2017). There were notable decreases in both free and secondary bile acids, such as deoxycholic acid and 12-keto-lithocholic acid, and SCFAs, like butyric and pivalic acids. These metabolic shifts were closely linked to race performance and the athletes’ training histories. This demonstrates that while the GM composition might remain unchanged in highly trained individuals, its metabolic functions, primarily related to bile, fatty and amino acid metabolism, are dynamically responsive to physical stress. As summarized in Table 3, these findings reinforce the nuanced interplay between exercise, gut microbial ecology, and host physiology, emphasizing the need for more comprehensive multiomics approaches (e.g. metatranscriptomics and metabolomics) to clarify the metabolic adaptability and restoration capacity of the GM under different exercise intensities and regimens.

**Table 3.** Thematic overview of exercise-induced GM changes in humans and potential implications for athletic performance.

Theme	Key observations	Representative supporting evidence
Lactate-metabolizing bacteria and performance	• *Veillonella* species proliferate postmarathon/ultramarathon, converting lactate to SCFAs (e.g. acetate, propionate) • *V. atypica* supplementation preserves or enhances exercise performance • *B. uniformis* supports endurance via hepatic glucose production	• Significant *Veillonella* increase after endurance races (Scheiman et al. 2019, Grosicki et al. 2019) • *V. atypica* supplementation study (Gross et al. 2023) • *B. uniformis* promoting endurance (Morita et al. 2023)
Role of gut archaea	• *M. smithii* prevalent in >95% of humans, especially in athletic or lean phenotypes • High methane metabolism influences SCFA dynamics and overall fermentative efficiency • Cross-feeding with fermenting bacteria can enhance energy extraction and maintain gut ecosystem stability	• *M. smithii* presence linked to increased diversity, observed in competitive cyclists (Dridi et al. 2009, Petersen et al. 2017) • Correlations with SCFA production, synergy with *Bacteroides thetaiotaomicron* (Catlett et al. 2022) • Association with lean BMI and weight loss (Ruaud et al. 2020, Mohr et al. 2024)
Extreme physical stress and allostatic load	• Ultraendurance events can trigger blooms of potentially pathogenic taxa • GI permeability and inflammation may increase under harsh physical/mental stress • Host resilience varies; not all athletes report GI distress	• Marathon/ultramarathon: ↑ potentially pathogenic microbes (Grosicki et al. 2019, Saragiotto et al. 2024) • Ski march study: ↑ alpha-diversity but higher GI permeability and inflammation (Karl et al. 2017)
Physical inactivity and GM changes	• Dry immersion and other severe hypoactivity models show shifts in GM composition such as increased *Clostridiales* and *Lachnospiraceae*, reduced fecal propionate	• Dry immersion: muscle atrophy, altered GM (Jollet et al. 2021)
Metabolic versus compositional resilience	• GM composition often remains relatively stable in highly trained individuals, but metabolic functions (e.g. bile acid, fatty acid, amino acid pathways) shift significantly • Changes in bile acid profiles and SCFAs correlate with performance metrics and training history	• Ironman triathletes: stable composition but altered metabolome, notably bile and fatty acids (Grosicki et al. 2023b) • Similar findings of stable composition but changed metabolites in Arctic ski march (Karl et al. 2017)

### The athletic GM: resilient and stable, yet challenged by stressors

While generally stable over time (Faith et al. 2013), the GM tends to be most unstable during sudden lifestyle changes or upon exposure to other microbiome-modifying factors. GM instability, in turn, may lead to, or be an indicator of, negative health outcomes. As discussed previously, multiple factors associated with exercise bouts at the acute scale may initially disrupt GM stability. Such factors may compound over time to increase microbial diversity and functional redundancy (Chatelier et al. 2013) potentially producing a GM with the ability to more readily restabilize its original state following exercise-induced perturbations. This functional redundancy allows microbial communities with similar roles to compensate for the loss of beneficial strains, maintaining ecosystem functions despite changes in community composition (Blakeley-Ruiz et al. 2019).

Resilience traits within the GM have implications for coping with the stress of exercise (Table 4), and how that stress may culminate over time to shift ecological states. Characteristics of an exercise-associated GM are shaped by a variety of internal and external stressors, both intermittent and continuous. However, directly confirming if an athletic GM shows increased resilience relative to the GM of other populations (which also tend to show resilience) is challenging, in part, because it is often not possible to expose the different populations to the same exercise-induced stressors. This challenge also extends to determining the extent to which exercise modulates the resilience threshold of the GM. Another important issue is that GM resilience can be a double-edged sword. A dysbiotic but resilient GM is undesirable and can be difficult to revert to a health-associated state. Such resilience may be linked to an increased allostatic load, reflecting a potential trade-off in the holo-genome’s fitness. This highlights that resilience alone does not necessarily confer health and performance benefits; a highly diverse and functional microbiota is also essential. In line with this hypothesis, Mach et al. (2020) studied elite sport horses exhibiting high levels of apathy and chronic stress due to continuous physical and emotional pressures from competitions and intensive training. Findings demonstrated that these horses developed a highly resilient microbiota that was not beneficial and could not be reversed even when exposed to positive environmental challenges, such as a temporary period in a more natural environment (i.e. out to pasture with conspecifics) (Mach et al. 2021a). Hence, the acquisition of a gut ecosystem that has a high resilience potential may make the management of interventions to reshape the community difficult.

**Table 4.** Proposed key traits of exercise-associated GM resilience.

Taxonomic diversity	• Enhanced diversity in athletes suggests a robust ecosystem capable of resisting pathogens and adapting to environmental stressors, improving health and performance stability.
Functional diversity	• High functional diversity means that the microbial community has a broader metabolic capacity to respond to and recover from physiological challenges. In athletes, this can translate into more efficient nutrient metabolism, enhanced energy production, and improved recovery processes after intense workouts. This functional adaptability under stress is a key aspect of resilience, as it ensures that the microbiome can maintain homeostasis and support the host’s health and performance under varying conditions.
Keystone taxa	• These taxa may support enhanced performance and recovery by stabilizing microbial communities against stressors associated with intense physical activity. They facilitate essential processes such as nutrient cycling, pathogen inhibition, and the maintenance of microbial diversity, ensuring the microbiome’s ability to adapt and respond to environmental and physiological changes.
Metabolic products	• SCFAs such as butyrate, propionate, and acetate play a role in maintaining gut barrier integrity, modulating inflammation, and providing energy to colon cells. In athletes, increased production of these products can enhance energy metabolism and recovery processes, contributing to a more resilient GM capable of quickly adapting to the stresses induced by intense physical activity. • Adaptive shifts in bile acid profiles in athletes support increased energy demands and recovery, influencing the gut environment and fostering resilience against exercise-induced changes. • Fatty acid amides stimulate sensory neurons in the gut that link to the brain’s reward system, potentially enhancing the desire and capacity for exercise by increasing dopamine levels during physical activity.
Metabolic flexibility	• The GM’s ability to adjust metabolic pathways is crucial for optimizing energy production and nutrient absorption, supporting endurance and recovery.
Stress response genes	• The presence of microbial genes that are activated during physical stress can indicate a microbiome’s readiness to handle and adapt to the stressors associated with intense exercise, contributing to the overall resilience of the GM.

Better understanding of conditioning roles (specific functions that certain microbes perform to adapt and modulate the gut environment), such as those exhibited by individual microbes like *Veillonella* (Scheiman et al. 2019) and *Akkermansia* (Fernández et al. 2021), appear to be critical for understanding traits of GM resilience. These keystone microbes exemplify the adaptive capabilities of the GM in response to the physiological demands of regular physical activity. In addition, microbes within the GM often display cooperative behaviors to stay competitive. For instance, bacteria use quorum sensing via signaling molecules called autoinducers to monitor and respond to cell density, environmental conditions, and community composition. This communication system enables microbes to collectively adjust activities based on the surrounding environment (Dejea et al. 2014). Even seemingly less fit bacterial species may carry genomic adaptations that enhance their survival in environments scarce in micronutrients, thereby increasing their fitness (Mach and Clark 2017). Additionally, dynamics such as progressive amensalism or colonization resistance can influence community structure by enabling dominant species to inhibit the growth of other taxa, thus maintaining ecological balance. Thus, shifting to another state may require a level of depletion of the keystone, conditioning microbial features to allow others to colonize, flourish, and support a distinct network synergy as suggested by studies showing shifts in the *Bacteroides*/*Prevotella* ratio in response to physical activity in rodents (Lambert et al. 2015).

In relation to conditioning roles, host-associated factors exert substantial influence over the GM’s responsiveness to exercise including when physical activity is initiated in a lifespan. For example, exercise in young animal models showed increased alpha-diversity, *Bifidobacteria* and methanogens abundance, and lean body mass, which persisted after stopping exercise, suggesting early adaptations (Mika et al. 2015). This period of developmental plasticity in young hosts mirrors a similar susceptibility to change in their developing microbial ecosystems. In humans, early exercise in children with obesity shifted microbiota profiles toward healthier states (Quiroga et al. 2020), while adult microbiota shows resistance to long-term changes, even with increased exercise (∼40%) and especially in disease states (Knoll et al. 2023). This suggests that early interventions might facilitate fostering a beneficial microbial state that could have lasting health impacts.

Additional host factors also significantly determine the GM’s resistance and resilience to changes. For example, Allen et al. (2018b) discovered that previously sedentary, lean, and obese adults responded differently to short-term endurance exercises. While lean participants experienced increases in fecal concentrations of SCFAs, individuals with obesity did not show these changes (Allen et al. 2018b). This difference illustrates how the sedentary GM may possess inherent resilience that prevents substantial microbial shifts without prolonged or intense exercise stimuli. Supporting these findings, even though acute and short-term exercise typically shows limited impact on microbial diversity in highly fit individuals (Grosicki et al. 2023b, Zhao et al. 2018, Tabone et al. 2021, Sato and Suzuki 2022, Fernandez-Sanjurjo et al. 2024), it can lead to rapid metabolic adaptations that enhance performance and facilitate recovery, including notable changes in fecal bile acid and fatty acid profiles (Grosicki et al. 2023b), organic acids (Zhao et al. 2018), and tryptophan, tyrosine, and phenylalanine (Tabone et al. 2021). Such changes have implications for suppressing fatigue (Liu et al. 2017) and increasing bacterial fitness, as tryptophan is vital for survival and its biosynthesis is generally ubiquitous across taxa (Merino et al. 2008). Collectively, these studies suggest that even if exercise stress on a short time-scale has little impact on GM diversity, the GM can rapidly respond with metabolic outputs that could impact certain taxa and host functions.

Overall, the dynamics of an exercise-associated GM and its resilience can be categorized into two main areas: host control and GM characteristics. Host control, involves selective pressures such as genetic background, metabolism, nutrient provision, immune tolerance, the protective function of the mucus layer, mitochondria–microbe interactions, peristaltic movements, and redox status regulation. These factors are crucial in determining, which microbial species thrive or diminish, thereby shaping the GM’s composition and functionality. GM characteristics include microbial diversity, functional redundancy, metabolic output and flexibility, microbial interactions, and colonization persistence. These elements enable the GM to withstand and adapt to the physiological and environmental stressors of an active lifestyle. Future studies probing GM resilience on multiple time-scales may enable microbiota-targeted strategies to mitigate high physical and emotional stress in athletes. Moreover, more nuanced resilience metrics—including metagenetic diversity, gene flow, and interkingdom interactions—may better capture ecosystem stability and recovery than traditional diversity indices alone. Continued research is necessary to unravel the complexities of the GM in active individuals, focusing on identifying patterns of stability and resilience and understanding how short-term responses to exercise stress inform long-term health implications.

## Individual factors contribute to GM response heterogeneity

Despite numerous studies, significant discrepancies remain in the reported GM modifications following exercise, reflecting the complexity and variability of individual responses. While acute and short-term stressors might not always prompt substantial shifts in a resilient adult GM (Grosicki et al. 2023a), the cumulative effects of these stressors, especially when combined with dietary adjustments, could progressively mold a GM characteristic of those observed in athletic populations. The longitudinal adaptation of previously sedentary individuals to regular physical activity and the corresponding physiological and microbiome changes, however, remains an underresearched area. A case study by Barton et al. (2021) tracked two unfit males over 6 months of progressive exercise training, documenting biweekly changes in the GM. The study observed increases in GM alpha-diversity after ∼18 weeks of training, suggesting a need for consistent exercise over a prolonged time period to achieve sustained enhancements in microbial diversity. Notably, exercise-induced taxonomic shifts in the GM were highly personalized, suggesting that the pretraining GM along with the host genome, its genetic and epigenetic variants, and its expression can contribute to phenotypic variation in the GM and the ability of the GM to cope with varying environmental pressures.

An intriguing question arises (modified from Olbricht et al. 2022) regarding the dynamics of these microbial communities in the context of exercise-induced modification: Are exercise-associated microbiota growth-limited or colonization-limited? This question delves into understanding whether all microbes are inherently present in all individuals, awaiting conducive conditions for growth, or if bacterial colonization events primarily constrain the GM’s composition. Scheiman et al.’s (2019) research suggests that the high-lactate environment of athletes provides a selective advantage for the growth of lactate-metabolizing organisms such as *Veillonella*. However, exercise-induced effects on the GM may depend on baseline composition (Bycura et al. 2021, Mach et al. 2022), implying that GMs are likely colonization-limited, with the existing microbial community playing a crucial role in shaping the response to environmental changes. Thus, the introduction of exogenous microbes as a strategy to foster microbial adaptation is an emerging field (i.e. strains of *Veillonella*; Gross et al. 2023) garnering interest as a means to manipulate the microbiome for exercise performance benefits.

A vital aspect of this dynamic is understanding the difference between responders and nonresponders to exercise interventions. For example, a study on untrained individuals revealed that GM composition could predict the efficacy of exercise regimens in enhancing both resistance and cardiovascular fitness, with specific microbial taxa correlating differently with each exercise modality (Bycura et al. 2021). Similarly, some individuals may not respond or may even react adversely to exercise in terms of insulin sensitivity and glucose homeostasis (Böhm et al. 2016). That variability may be related to GM functional changes during exercise interventions independent of major shifts in community structure. For instance, a 12-week study involving overweight men with prediabetes demonstrated that exercise responders experienced enhanced SCFA production and branched-chain amino acid breakdown, while nonresponders exhibited increased levels of metabolites like indole and *p*-cresol, suggesting distinct metabolic processing (Liu et al. 2020). Notably, fecal microbial transplantation from exercise responders alleviated insulin resistance in obese mice, indicating that functional microbiota changes are crucial in mediating exercise benefits. In subsequent research using samples from Liu et al. (2020), proteomic analysis differentiated between responders and nonresponders, revealing that responders exhibited activation of metabolism-related proteins and suppression of inflammatory markers, whereas nonresponders showed increased stress-related proteins (Diaz-Canestro et al. 2023). Furthermore, proteins like trefoil factor 2, positively associated with changes in insulin resistance and fasting insulin and GI mucosal immunity, were increased in nonresponders, suggesting modulation of the microbiota via regulating genes involved in innate host antimicrobial defense (Baus-Loncar et al. 2005).

These insights reveal the complexity behind the responder and nonresponder dichotomy, suggesting that while exercise may impact health and the GM, the nature and extent of these effects are highly individualized and depend, in part, on the initial GM. Indeed, the stability of individual species, genes, and transcripts over time is generally correlated with average baseline relative abundance and prevalence (Mehta et al. 2018), but also depends on the host’s genetic make-up and epigenetic factors. Admittedly, this is not always the case, as in the example of *M. smithii*, which, despite their stable presence, appear to be dominant contributors to the expression of unstable genes, suggesting specialized roles associated with targeted, highly variable activity over time (Mehta et al. 2018). Regardless, it is not merely the presence of certain microbes that matters in the context of exercise–GM interactions, but the functional potential of the GM and the interaction with host genome, metabolism, and immune function.

## Toward improved understanding of GM resilience and stability in response to exercise

### Capturing temporal dynamics to model potential microbiota configurations

**Figure 4.**
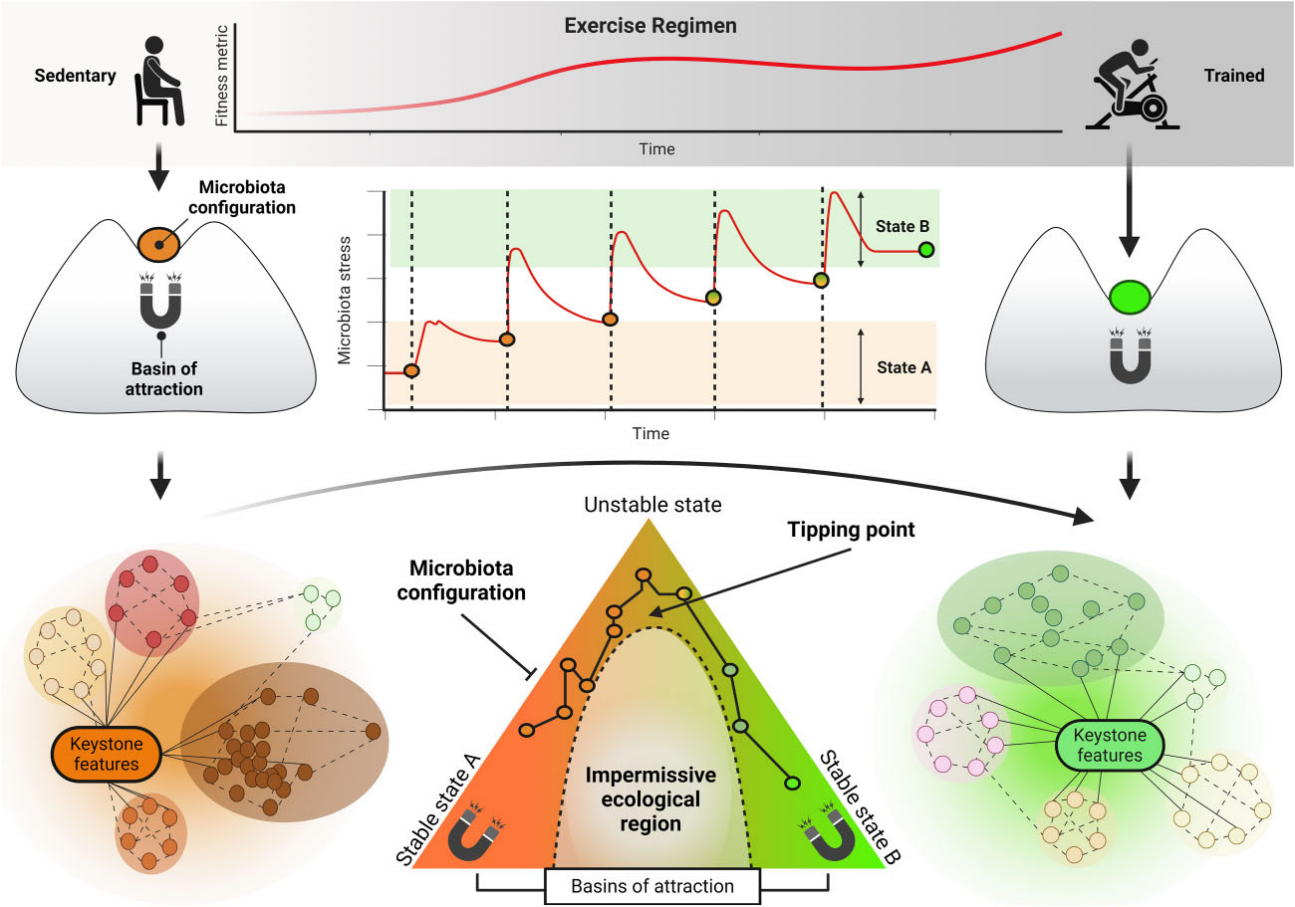
Dynamic shifts in GM across different exercise states. Conceptualization on how sustained exercise stress influences the GM, leading to shifts in ecological states over time. Contrasting alternative stable states in sedentary versus exercise-trained individuals, exercise can push the GM across ecological thresholds or tipping points, resulting in a transition to a new stable state. These transitions are influenced by basins of attraction—keystone features like dominant taxa or specific microbial community structures. Not only do these basins dictate community composition and diversity, but they also contribute to the stability and resilience of these states, reflecting the allostatic load of the holobiont and its management through exercise. Created in BioRender.com.

To deepen understanding of how consistent exercise shapes the GM over time, comprehensive longitudinal studies that capture the nuanced temporal shifts induced by regular exercise are needed. The concept of accumulated stress suggests that even small, regular stimulations can cumulatively drive significant shifts in GM community dynamics when applied intermittently or continuously (Fig. 4). This is particularly relevant when considering stochastic dynamical systems, which posits that alternative stable states in an ecosystem are often separated by an unstable intermediate, known as a tipping point (Lahti et al. 2014). Here, minor perturbations can catalyse a rapid and self-sustaining transition to a new state (Guchte et al. 2020). The notion of tipping elements within the GM suggests that specific functional components or taxa may act as critical indicators or facilitators of these transitions, reflecting broader shifts in ecosystem stability and host physiology (Lahti et al. 2014). Modeling these dynamics could illuminate the conditions under which the GM exhibits resilience or vulnerability, highlighting how specific microbial taxa or community structures play a pivotal role in maintaining community stability or dictating shifts in composition. Predicting and influencing these microbiota transitions from state to state may involve using probability densities of bacterial abundance to estimate basins of attraction, which might indicate under what conditions the microbiome is most resilient or susceptible to change (Levy et al. 2020). Indeed, to shift a GM to a configuration, one may need to overcome substantial ecological gradients or barriers into areas of permissivity (population bottleneck in which both are relatively depleted) based on exclusionary occurrence across intestinal habitats (Zhang et al. 2016). Identifying these ecological gradients or barriers that need to be overcome to transition between states has tremendous implications for “engineering” the GM. For example, diminishing certain features or clusters of features to make the ecological landscape conducive for desired features.

In addition, exploring cooccurrence relationships, community network structures, and functional outputs will provide a more systemic understanding of how the entire GM assembles and remains or moves to alternative stable states in response to exercise training. Identifying microbial tipping points requires a nuanced approach, considering not just the presence or abundance of certain microbes but also the complex interplay of host factors, environmental exposures, and intrinsic microbial community dynamics. As a proof-of-concept, Shaw et al. (2019) utilized stability landscape analyses to demonstrate how the human GM, conceptualized as resting in the equilibrium of a stability landscape of all possible states, can be perturbed into alternative states by interventions like antibiotics. By modeling the microbiome's stability landscape (via analytical impulse-modeling of alpha-diversity), they could predict recovery or transitions postantibiotic treatment, offering insights into how to potentially guide the microbiome back to health or maintain its resilience against disturbances. However, to our knowledge, similar approaches have not been used in the context of exercise stress. Moreover, it is crucial to recognize that while such models provide valuable insights, they simplify the microbiome’s incredibly complex and dynamic nature. Each microbial community is more than the sum of its parts, with intricate interactions and dependencies that influence its function and stability. Therefore, while stability landscape frameworks and impulse–response models are powerful tools, they are just starting points for understanding the multifaceted dynamics of the GM in the context of exercise and other interventions.

Generalized Lotka–Volterra (gLV) models and associated variants could further enhance our understanding by describing changes over time in a population of species based on their intrinsic growth rates and interactions (Gonze et al. 2018). These models can capture several commonly encountered network structures, including food chains, modularity, scale-freeness, and small-world networks. They provide valuable insights into the ecological dynamics within the gut, potentially identifying keystone species that significantly influence community composition and stability and those that impact health, such as by inhibiting pathogens. However, gLV models primarily focus on pairwise interactions and assume that they are additive, which may not capture the full complexity of microbial interactions that are often nonlinear and influenced by a range of biotic and abiotic factors. Moreover, these models typically do not account for immigration or emigration events within the community (assuming a closed system), which might not reflect the dynamic nature of microbial ecosystems. The assumption of homogeneity in populations is another limitation. In reality, microbial communities are incredibly diverse, with variations in genetic and functional attributes within species. Despite these limitations, gLV models are still valuable for making broad predictions about factors governing microbial community stability and dynamics. They provide a framework to understand how each species’ abundance changes over time and how other community members influence these changes. By learning from these models, researchers can identify critical community members and interactions essential for maintaining a healthy and stable microbial ecosystem or contributing to its perturbation.

Understanding these dynamic community states is crucial for identifying when subtle shifts in exercise routines, dietary patterns, or other factors push the microbiome from one stable state to another, potentially leading to variations in health outcomes or physical performance. Many factors associated with exercise bouts at the acute scale may initially shift GM stability, including hyperthermia, change in blood flow, inflammation, hydration status, and kinetics of the GI tract. Other important considerations are related to the confounding host factors, such as genetics, diet, sleep, medication use, health status, stress, and environmental exposures. This can further feedback to the ecosystem level, as the resulting changes in the community composition may influence the GI processes and environment.

### The inseparable dynamic of diet

Diet is fundamental in shaping microbiota composition and metabolic outputs (Sonnenburg et al. 2016). Ecologically, diet exerts strong selective pressures on gut microbial communities (Byndloss et al. 2018), driven by competition for nutrients and increased fitness through cross-feeding interactions (Morris et al. 2013). Leveraging diet for improving the mediatory mechanisms of the GM on athletic performance has been the topic of recent, detailed reviews (Hughes and Holscher 2021). Briefly, complex carbohydrate metabolism, performed by gut microbes, seems crucial, as elite athletes often derive much of their energy intake from poly- and monosaccharides (Mach and Fuster-Botella 2017, Murtaza et al. 2019). Endurance athletes are often advised to consume higher energy and carbohydrate diets, often with a lower absolute fiber load (in soluble and insoluble forms), impacting transit time and GM turnover. Complex carbohydrates are particularly beneficial, increasing the production of SCFAs like butyrate. From a fermentation standpoint, the type of substrate an athlete supplies to the colon governs the physicochemical environment that, in turn, shapes microbial ecology. Microbiota-accessible carbohydrates are rapidly fermented, lowering luminal pH and favoring butyrate-producing commensals; many taxa thrive only if they can also tolerate this acidified milieu (Hughes and Holscher 2021). A consistently acidic colonic environment supports SCFA production, reinforces tight-junction expression (Blachier et al. 2017), and suppresses opportunistic pathogens and proteolytic bacteria (Zhang et al. 2020). When fermentable fiber is scarce and protein intake dominates, metabolism shifts toward amino-acid catabolism, raising pH and osmolarity and generating ammonia, phenolic compounds, and *p*-cresol that can erode the mucus layer and compromise barrier integrity (Mancin et al. 2025). Thus, high-carbohydrate/adequate-fiber diets not only supply substrate for SCFA synthesis but also create a biochemical landscape that selects for health-promoting taxa, whereas low-fiber, high-protein patterns can tilt the environment toward potentially deleterious proteolytic fermentation (Mancin et al. 2025). For example, Furber et al. (2022) compared the effects of high-protein and high-carbohydrate diets on the GM of 16 highly trained endurance runners. Findings revealed that a high-protein diet led to performance declines and significant shifts in the gut phageome. This included reductions in bacteriophage diversity and changes in Sk1virus and *Leuconostoc* populations. In contrast, the high-carbohydrate diet enhanced performance and promoted populations adept at carbohydrate fermentation and metabolism. Notably, athletes with a stable GM across both the high-carbohydrate and high-protein interventions typically showed superior performance, highlighting the interplay between diet, microbial adaptation and resilience, and athletic output. Athletes who did not respond well to the high-carbohydrate diet showed a sustained loss of bacteriophage functional richness even after the dietary intervention ended, indicating a potential connection between a lack of functional microbial plasticity and suboptimal performance. This contrasted with the observed expansion of viral communities in the high-protein diet group, possibly reflecting increased bacterial stress due to a scarcity of fermentable carbohydrates.

As highlighted by Furber et al. (2022), the dietary patterns of athletes and physically active individuals, which generally diverge from those of less active populations, merit consideration when assessing the effects of exercise training on the GM. This includes the common use of dietary supplements such as probiotics, prebiotics, postbiotics, and other GM-modulating agents among athletes (Jäger et al. 2019, Hughes and Holscher 2021). Recent controlled research suggests that probiotic supplementation can complement diet-driven modulation of the GM in sport. A recent scoping review of 45 randomized trials in athletes and physically active adults reported wide variation in strains, doses (10^8^–10^11^ CFU/day) and intervention lengths, yet found generally low risk of adverse events and frequent assessment of GI or immunity-related outcomes (Mohr et al. 2022b). A 2023 systematic review of 13 RCTs concluded that certain single-strain or multistrain probiotics improved endurance–performance metrics and reduced fatigue and muscle pain (Dio et al. 2023). Parallel evidence shows reductions in upper-respiratory and GI illness incidence, largely via immune modulation and barrier support (Díaz-Jiménez et al. 2021). The International Society of Sports Nutrition position stand further stresses that probiotic benefits are strain- and dose-dependent and highlights mechanisms—such as enhanced gut–barrier integrity and increased amino-acid absorption—that are directly relevant to recovery and performance (Jäger et al. 2019). Collectively, these findings indicate that evidence-based, strain-verified probiotic strategies may augment athlete health and, in certain contexts, performance.

Beyond probiotics, accumulating work indicates that diet itself may have a stronger influence on the GM than exercise alone. For instance, Cronin et al. (2018) observed that increased protein consumption, either alone or combined with exercise, had a more substantial effect on shaping the virome over 8 weeks than exercise alone in previously sedentary humans. Similarly, Yun et al. (2022) reported that diet had a more pronounced effect on gut microbial richness and diversity than exercise in adult mice, indicating that dietary factors may drive more significant changes in the GM than physical activity. In a similar way, the gut community microbial structure observed in endurance horses was primarily influenced by diet, with host properties, exercise distance, and exercise effort showing minimal impact (Plancade et al. 2019). Moreover, early-life diet was found to have a lasting impact on the GM, reducing bacterial diversity in selectively bred exercise-adapted mice (76 generations of exercise) (McNamara et al. 2021). Therefore, the intricate interplay between diet and the GM likely influences GM responses to exercise and GM impacts on athletic outcomes. Understanding and harnessing this dynamic relationship will be important for optimizing health and performance, making it a critical area of focus for future research and practical applications in sports and exercise nutrition.

### Research priorities for assessing resilience in exercise-associated GM

Focused research directions to enhance our understanding of resilience within the exercise-associated GM are outlined in Table 5. Robust longitudinal studies are crucial for assessing the long-term impacts of different exercise modalities and intensities on the GM. In these studies, dietary intake should be carefully tracked or controlled to reduce the confounding influence of varying nutrient patterns on GM shifts. These studies should document temporal microbiota composition and functional changes during various training phases, including intense activity and subsequent recovery periods, to determine if exercise-induced shifts are transient or lead to stable alterations. Indeed, it is likely that increased alpha-diversity observed in athlete GMs relative to less active populations occurs over a longer time scales and/or may be apt to increase in less fit and younger individuals. We suggest that the gut microbial diversity observed in athletes is likely the result of the coevolution between microbial ecosystems and their hosts, where a top-down selection favors gut ecosystems with stable communities and a high degree of functional redundancy, and bottom-up selection drives microbial cells to become functionally specialized (Ley et al. 2006).

**Table 5.** Future research directions in exercise-associated GM resilience and functionality.

Area of research	Goals and methods	Implications
Longitudinal studies	Conduct studies with dense sampling in both sedentary and physically active populations to observe long-term effects of various exercise regimens on the GM, capturing temporal dynamics during intense activity and recovery and their relation to changes in health and performance.	Identify tipping points and time courses of change. Determine if exercise-induced changes are transient or lead to stable alterations in the GM.
Resilience assessments	Assess resilience to nonexercise stressors (i.e. nutritional challenges, antibiotics, infectious disease, and so on) in active versus sedentary individuals.	Determine if features of GM resilience are differentially influenced in an exercise-associated GM and agnostic to the destabilizing perturbation.
Sectional GI tract analysis	Implement methods to analyse microbial communities and physiological responses along different sections of the GI tract, moving beyond fecal samples to better understand local variations and their implications for health.	Provide a more accurate picture of gut health in athletes, informing targeted interventions and dietary strategies.
Mechanistic studies	Elucidate the biochemical pathways and microbial metabolites influenced by exercise, and identify their links to metabolic and immune responses.	Highlight potential therapeutic targets to enhance gut health and systemic resilience.
Diet, supplementation, and exercise interplay	Examine how dietary strategies and supplementation can enhance exercise-induced GM adaptations, focusing on diets and supplements that boost microbial diversity and microbial functions linked to health and performance.	Optimize GM modulation and maximize health benefits of exercise through targeted nutritional approaches.
Personalized approaches in sport science	Explore individual variability in exercise response due to GM, genetic, metabolic, lifestyle factors, age, sex, and race/ethnicity to personalize exercise recommendations.	Tailor exercise protocols to maximize microbially associated health benefits, enhancing personalized health care.
Biomarker development	Develop noninvasive biomarkers for real-time monitoring of GM changes and related health outcomes influenced by exercise.	Provide tools for ongoing assessment of gut health and resilience, supporting informed health decisions.
Resistance training	Investigate the effects of resistance training on GM, particularly regarding muscle mass, metabolism, and aging-related health issues.	Extend understanding of exercise types beyond endurance, offering broader insights into exercise impacts.
Gut–brain axis	Study how exercise-induced GM changes affect the gut–brain axis, focusing on microbial metabolites that influence brain function, mental health, cognitive function, and stress resilience.	Unveil mechanisms by which physical activity can influence psychological and GI functions.
Environmental factors	Assess the impact of environmental conditions such as heat, cold, elevation, and pollution on exercise-induced changes in the GM.	Understand how external stressors affect GM adaptation and resilience, informing training in varied climates.
State dynamics	Investigate the GM during states of detraining and retraining to understand microbial transitions and their functional implications.	Explore how fluctuations in training intensity influence GM stability and resilience.
Injury recovery and rehabilitation	Determine how exercise-induced changes in GM can influence. inflammation, tissue repair, and recovery times in athletes.	Gain a deeper understanding of the complex interactions between exercise, GM, overall health, ultimately leading to optimized strategies for enhancing athletic performance.
Travel and competition environment	• Longitudinal sampling before, during, and after travel across time-zones, altitudes, or climates. • Record sleep/circadian markers, diet logs, hydration status, stress hormones, stool pH/consistency, and pathogen screening. • Use multiomics (metagenomics and metabolomics) and wearable tech to link circadian disruption, dietary change, and environmental exposures to GM dynamics.	Clarify how jet lag, altered food/water hygiene, and location-specific microbes influence gut-microbiota composition, resilience, and athlete performance; inform mitigation strategies (e.g. timed light exposure, targeted pre/probiotics, and phased diet adjustments).
Holistic approach	Integrating the study of respiratory microbiota with mucins, and GM research provides a more comprehensive understanding of the microbiota’s role in overall health and performance. Athletes often experience high respiratory demands, especially during intense training and competition.	This holistic approach can lead to more effective interventions and personalized strategies for athletes.
Microbial engineering	The study of microbial engineering of athlete’s microbial communities.	Optimize nutrient absorption, boost immune function, and improve metabolic efficiency.

Abbreviations: GI, gastrointestinal; GM, gut microbiota.

Further investigations are needed to compare active individuals directly to other populations, using detailed longitudinal designs that capture a wide array of ecological metrics such as stability, plasticity, resistance, resilience, and community variability. While many studies reveal clear GM distinctions between athletes and sedentary controls, it is noteworthy that a recent, large-scale pooled metagenomic analysis did not reveal “sport-specific” signatures (Fontana et al. 2023). This reinforces the concept that, despite regular training and shared exercise stress, additional interindividual variables (e.g. genetics, dietary patterns, lifestyle, and training load) may, in some studies, obscure discipline-specific clustering. Consequently, the proposed model of exercise-induced gut microbial resilience should be viewed as a broad framework, rather than an absolute predictor of clustering by athletic discipline. The lack of distinct sport-based clustering supports the need for more uniform, controlled cohorts, and multiomics approaches to identify finer-scale microbial adaptations that might otherwise be masked by confounding factors. These studies should also consider the biochemical pathways affected by physical activity and account for individual variability (i.e. genetics and epigenetics) in exercise response. It is also critical to leverage and develop methodologies that capture microbial communities along the entire GI tract, not just from fecal samples. The prevalence of GI symptoms among athletes, for instance, highlights the limitations of using fecal samples to infer gut health, as these symptoms may reflect localized disturbances not captured by fecal microbial profiles. Thus, caution is warranted in interpreting these profiles as comprehensive indicators of gut health.

Additionally, most research related to the exercise-associated GM is related to endurance-type physical activity. Resistance training offers opportunity to address other important research questions, especially pertinent to muscle mass accretion and thus offsetting cardiometabolic derangement and the sarcopenic effects of aging. For example, recent research has provided evidence that a 6-week resistance training exercise program (3 days/week) in sedentary adults with overweight and obesity can increase GM diversity, SCFA-producing taxa, and microbial pathways related to carbohydrate metabolism and cell motility compared to sedentary controls, paralleling improvements in glucose metabolism (Cullen et al. 2024). Thus, investigating the mechanisms underlying the resilience of the GM in response to different types of exercise is crucial. Those studies should focus on how microbial communities adapt and maintain stability under physical and emotional stress to identify key factors that contribute to resilience. Additionally, determining how specific dietary components and supplements can enhance microbial resilience and overall health in athletes could lead to optimized and, perhaps, personalized dietary recommendations. Finally, environmental factors such as dietary intake and demographic variables like age, sex, stress loads, and race/ethnicity are crucial in shaping host–GM dynamics in response to exercise. Elite competitors also face repeated long-haul travel, rapid time-zone shifts, and abrupt changes in altitude, climate, and local food–water hygiene—stressors that can transiently disrupt circadian rhythms, immune tone, and gut–microbiota stability. By understanding these factors, we may eventually optimize health benefits of exercise and enhance athletic performance through GM-targeted strategies.

## Conclusion

The interaction between the host and the GM under the influence of exercise is complex and bidirectional, involving metabolic efficiency, immune regulation, and even endocrine functions. Regular physical activity serves as a hormetic stressor that not only challenges but also appears to enhance the diversity and functional capacity of the microbial ecosystem, which aligns with allostatic principles. These effects are likely mediated by exercise-induced changes in immune activation, thermal regulation, metabolism, and alterations in hydration status and oxidative stress that collectively drive the long-term adaptation of the GM. By fostering a resilient and diverse GM, consistent exercise may prepare this community to withstand and adapt to various physiological stresses, thus reducing allostatic load over time. Host-controlled factors like diet, training regimens, and lifestyle choices also significantly shape these dynamics, underscoring the interconnectedness of lifestyle, exercise, and gut health. Ultimately, by altering gut microbial communities, exercise has far-reaching and long-term impacts on the gut ecosystem and systemic health. As we continue to uncover the intricate relationships between exercise and the GM, it becomes increasingly clear that leveraging this knowledge could lead to innovative approaches in enhancing health and athletic performance through targeted microbial interventions. Harnessing a new-genome level understanding of individual microbial taxa, microbe–host and microbe–microbe interactions, and functional capacities that might operate at the microbial community level is essential to the success of resilience and ecological restoration following exercise.
